# CD133 initiates tumors, induces epithelial-mesenchymal transition and increases metastasis in pancreatic cancer

**DOI:** 10.18632/oncotarget.3228

**Published:** 2015-03-16

**Authors:** Alice Nomura, Sulagna Banerjee, Rohit Chugh, Vikas Dudeja, Masato Yamamoto, Selwyn M. Vickers, Ashok K. Saluja

**Affiliations:** ^1^ Division of Basic and Translational Research, Department of Surgery, University of Minnesota, Minneapolis, MN

**Keywords:** CD133, Pancreatic Cancer, invasion, metastasis, NF-kB

## Abstract

CD133 has been implicated as a cancer stem cell (CSC) surface marker in several malignancies including pancreatic cancer. However, the functional role of this surface glycoprotein in the cancer stem cell remains elusive. In this study, we determined that CD133 overexpression induced “stemness” properties in MIA-PaCa2 cells along with increased tumorigenicity, tumor progression, and metastasis *in vivo*. Additionally, CD133 expression induced epithelial-mesenchymal transition (EMT) and increased *in vitro* invasion. EMT induction and increased invasiveness were mediated by NF-κB activation, as inhibition of NF-κB mitigated these effects. This study showed that CD133 expression contributes to pancreatic cancer “stemness,” tumorigenicity, EMT induction, invasion, and metastasis.

## INTRODUCTION

Pancreatic cancer remains the only cancer type with a single-digit 5-year survival rate, a mere 6% [1]. Tumor recurrence, occurring in over 95% of pancreatic cancer patients, has been largely associated with the presence of cancer stem cells (CSCs) at these sites. Pancreatic cancer stem cells have been isolated and studied in multiple murine models since 2007 based on the expression of several markers present on the cell surface [2–5]. These included CD44, CD24, ESA, CD133, and c-Met, among others. However the biological significance of some of these markers have not been evaluated in pancreatic cancer.

CD133 (also known as prominin-1) is an established cancer stem cell marker in many cancers including pancreatic cancer. Many studies have demonstrated that expression of CD133 correlates with poor patient prognosis in pancreatic cancer, as well as other cancer types [2, 5, 6]. Thus far, not much is known as to what role CD133 plays in “stemness” and metastasis. CD133^+^ populations have shown increased tumorigenicity, self-renewal pathway signaling, and metastasis [3, 7, 8] as compared with CD133^−^ populations. Recent studies from our group have established that spontaneous LSL-Kras^G12D/+^;LSL-Trp53^R172H/+^;Pdx-1-Cre (KPC) mouse model of pancreatic cancer have ~7–8% CD133+ cells. CD133^+^ cells isolated from these tumors also showed increased NF-κB activity. Interestingly, CD133 expression in a number of pancreatic cancer cell lines also correlated with their invasiveness and migration potential when tested *in vitro* [7]. The KPC model of pancreatic cancer is a notoriously aggressive model with 100% penetrance and ~6 month survival [9]. High CD133 expression in these tumors thus seemed to be associated with poor prognosis of the cancer along with increased invasiveness [7].

Multiple studies done on colon cancer, liver cancer, gastric cancer, and neuroblastoma indicate that CD133 expression correlates to poor prognosis [2, 5, 6, 10–12], similar to what our studies showed in the KPC model. Studies regarding the link between the CSC population, epithelial-mesenchymal transition (EMT), and metastasis are still ambiguous. CSC populations have been shown to express markers of EMT and conversely, induction of EMT results in a more “stem-like” phenotype [13, 14]. Cells of the primary tumor undergo EMT, in which polarized, non-motile cells become highly motile, capable of local invasion and intravasation, resulting in distant metastatic colonization. EMT and metastasis have been reported to be regulated by the NF-κB signaling pathway in a number of cancers, including pancreatic cancer [15, 16]. Furthermore, NF-κB activation through IKK activity modulation leads to classical EMT marker changes and the promotion of cellular migration and invasion. In the context of pancreatic cancer, NF-κB activation has been shown to be absolutely essential for tumor development [17].

Though all of the above observations suggest that CD133 expression, invasion (and EMT), tumorigenesis and NF-κB activity have a linear relationship; previous studies have not yet shown this association. Based on our earlier observation that CD133^+^ cells from a KPC tumor are able to generate tumors at very low cell density [7], in the current study we overexpressed CD133 in MIA PaCa-2 cell line (having all other *cancer* phenotype, but extremely low CD133 expression) to generate a system in which we can study the downstream *effect* of CD133 surface expression and how its expression contributes to the cancer stem cell phenotype. Our study shows that CD133 expression in a cell line with very low endogenous expression of CD133 leads to increased tumorigenicity, tumor progression, and metastasis *in vivo*. Additionally, we show that induction of EMT and increased invasion by CD133 expression is mediated by NF-κB activation. This is the first report to demonstrate the functional role of the CSC surface marker CD133, and how it may contribute to the CSC phenotype in pancreatic cancer.

## RESULTS

### Expression of the cancer stem cell marker CD133 results in increased “stemness”

Our previous study showed that CD133^+^ cells from a KPC tumor were able to initiate tumors at very low cell density. To establish if CD133 expression influenced tumor-initiating property of a cancer cell, we used the pancreatic cancer cell line MIA PaCa-2 (with 0.1% endogenous CD133 expression) and overexpressed CD133. Overexpression of CD133 was confirmed by flow cytometry and by RNA expression (Supplementary Figure 1A and 1B).

**Figure 1:**
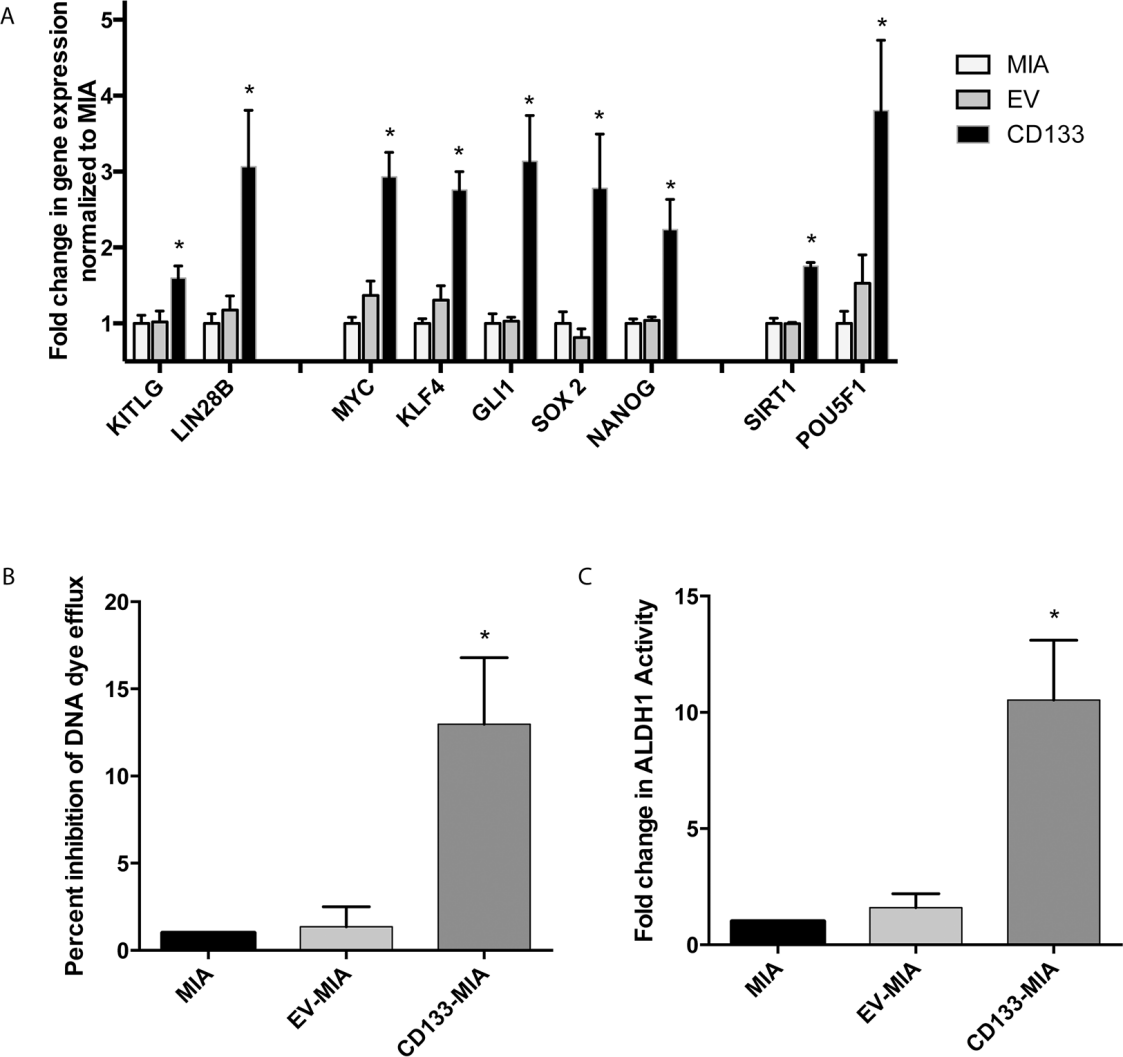
Expression of the cancer stem cell marker CD133 results in increased “stemness” CD133 overexpression led to **(A)** increased gene expression of “stemness” genes as compared with vector control **(B)** increased dye efflux and **(C)** increased ALDH activity.

CD133 overexpression led to an increase in cancer stem cell phenotype. Several genes associated with “stemness” were upregulated upon CD133 overexpression in MIA PaCa-2 cells overexpressing CD133 (CD133^hi^-MIA) as compared with Mia PaCa-2 and empty vector controls. KITLG (also known as stem cell factor) (1.60 ± 0.16 fold), LIN28B (3.06 ± 0.74 fold), MYC (2.93 ± 0.32 fold), KLF4 (2.76 ± 0.24 fold), GLI1 (3.14 ± 0.61 fold), SOX2 (2.78 ± 0.71 fold), NANOG (2.24 ± 0.40 fold), SIRT1 (1.75 ± 0.05 fold), and POU5F1 (3.80 ± 0.93 fold) (Figure 1A) gene expression were significantly upregulated upon overexpression of CD133.

In addition to these “stemness” genes, CD133 overexpression led to a 7.5 fold increase in ABCG2 gene and protein expression (Supplementary Figure 2A and 2D). Functionally, this increased cell drug transporter activity, as demonstrated by the dye efflux assay. CD133 overexpression increased dye efflux to 12 fold higher as compared with controls (Figure 1B). ALDH1 activity and dye efflux correlated with CD133 expression level (Supplementary Figure 5A and 5B). Interestingly, CXCR4 gene expression increased 8.9 fold (Supplementary Figure 2A) in CD133^hi^-MIA cells along with a 5.5% increase in CD133^+^CXCR4^+^ population (Supplementary Figure 2B and 2C). The CD24^+^CD44^+^ESA^+^ population increased by 7.9% (Supplementary Figure 2B) and ALDH activity within cells overexpressing CD133 also increased to 12% (Figure 1C).

### CD133 overexpression increased tumorigenicity

**Figure 2:**
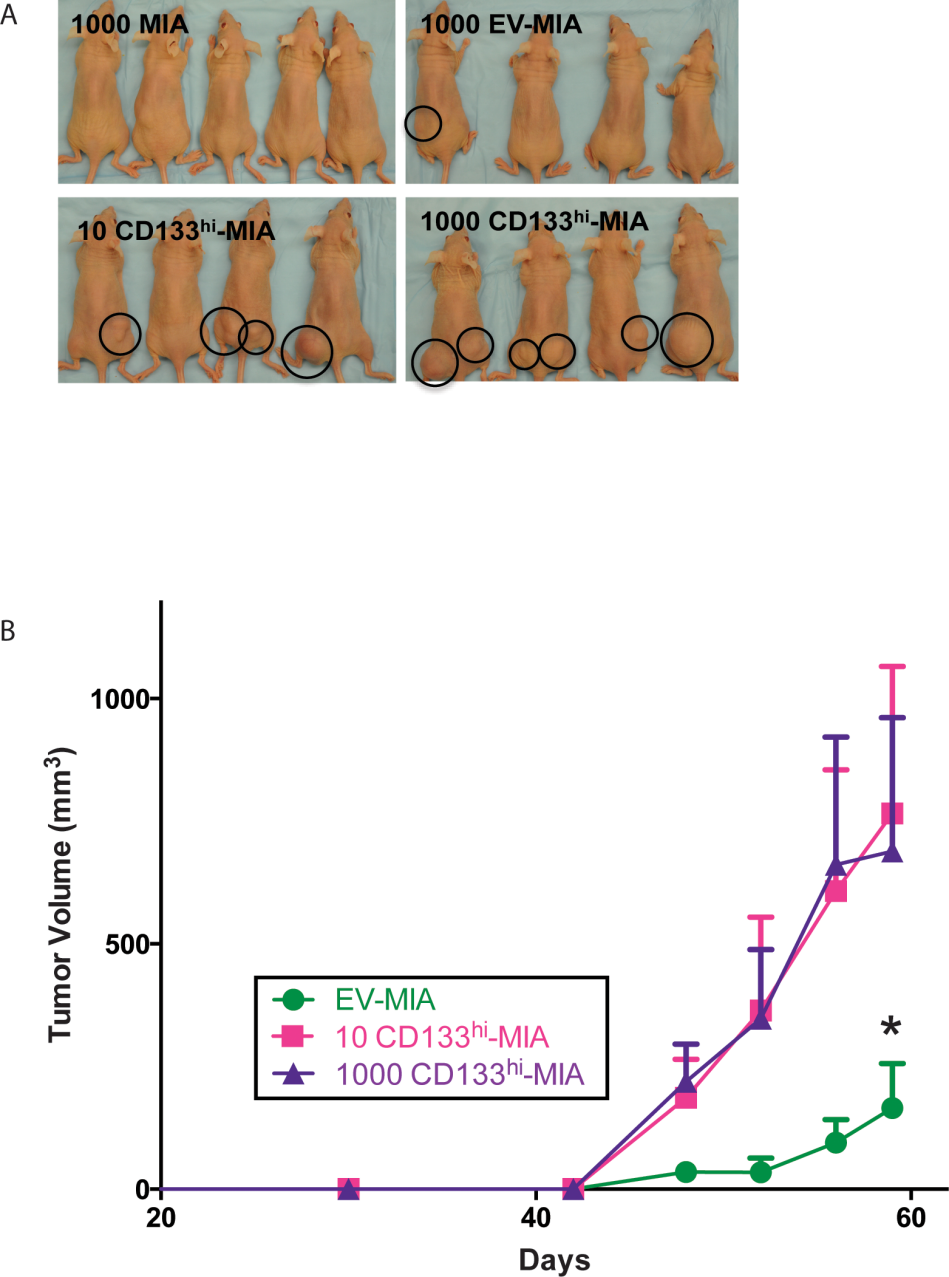
CD133 expression increases tumor formation **(A)** Representative images of tumor formation in MIA, EV-MIA, and CD133^hi^-MIA groups. **(B)** Tumor growth expressed in volume of tumors derived from EV-MIA and CD133^hi^-MIA groups.

To determine whether CD133 surface expression determined the ability of a cancer cell to initiate tumors, we overexpressed CD133 (CD133^hi^-MIA) and the empty vector plasmid (EV-MIA) in MIA-PaCa2 cells. Different numbers of these cells were injected subcutaneously into athymic mice. Only ten CD133^hi^-MIA cells were needed to form tumors in 7/16 mice or 1,000 cells in 10/16 mice within 53 days, as compared with the same number in MIA PaCa-2 (0/16) or EV-MIA (2/16) controls (Table 1 and Figure 2A).

**Table 1:** CD133 overexpression increased tumorigenicity

	Day 1	Day 53
1000 Mia PaCa2	0/10	0/10
1000 EV-MIA	0/16	2/16
10 CD133^hi^-MIA	0/16	7/16
1000 CD133^hi^-MIA	0/16	10/16

Tumors derived from 10 and 1,000 CD133^hi^-MIA cells showed an increased rate of tumor growth compared with those formed by the EV-MIA control cells. The average tumor volume of the CD133^hi^-MIA cells was 766.14 mm^3^ (10 cells) and 688.52 mm^3^ (1000 cells) compared to 165.27 mm^3^ from EV-MIA cells (Figure 2B). Surface expression of CD133 was 0.56% in EV-MIA and 21.1% in CD133^hi^-MIA cells, as determined by flow cytometry (Supplementary Figure 1).

### CD133 expression induces EMT and increases invasiveness *in vitro*

**Figure 3:**
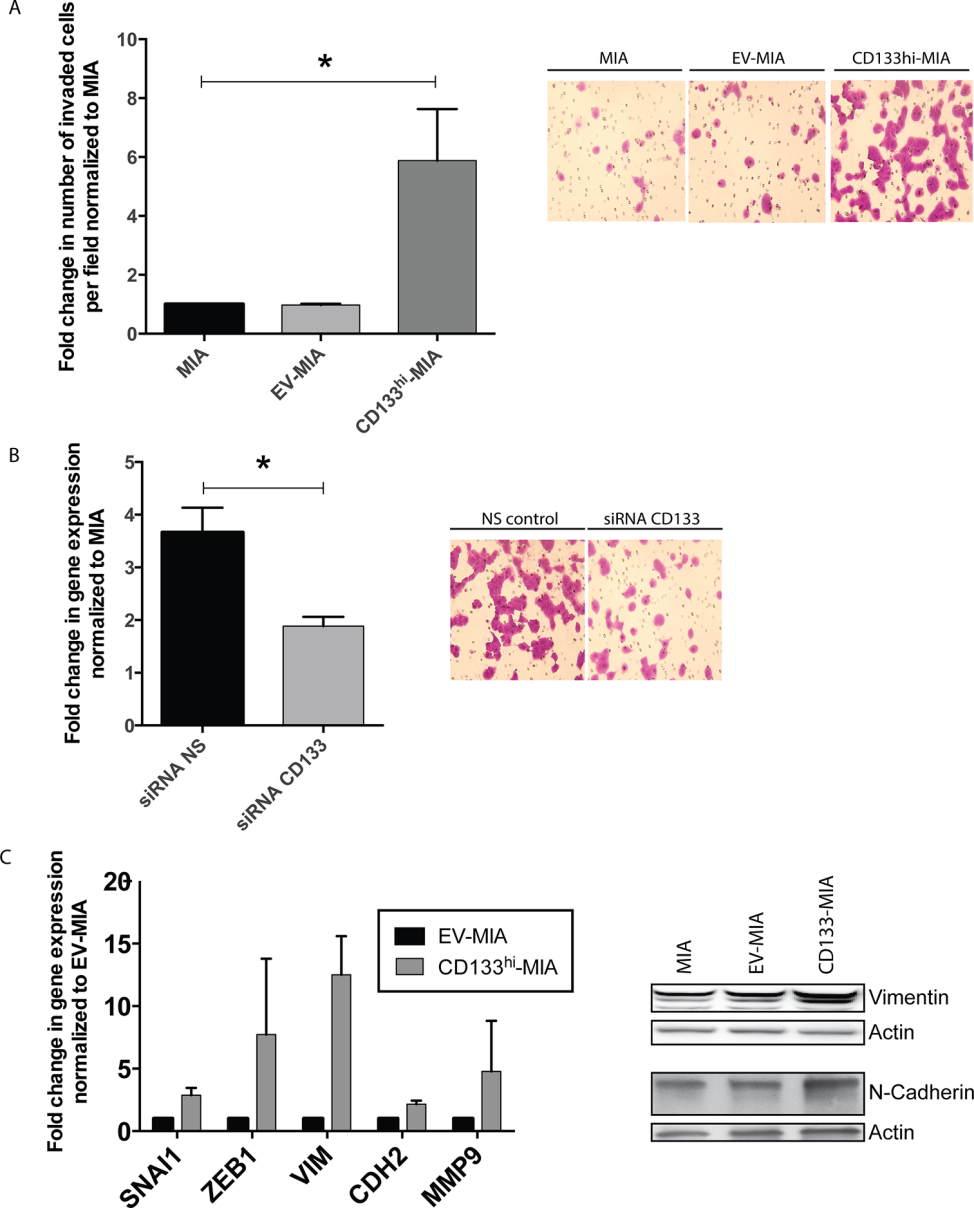
CD133 expression induces EMT and increases invasiveness *in vitro* **(A)** *In vitro* Boyden chamber invasion of MIA, EV-MIA, CD133^hi^-MIA with representative images from the Boyden chamber invasion assay, **(B)** Invasion upon CD133 silencing in CD133^hi^-MIA with representative images, and **(C)** EMT gene expression in CD133^hi^-MIA as compared with EV-MIA control and protein expression of Vimentin and N-Cadherin.

Our recent data indicated that CD133 expression correlated with the invasive ability of the cells. To determine if CD133 overexpression indeed led to increased invasiveness in MIA PaCa-2 cells, we performed a Boyden chamber invasion assay. The number of CD133^hi^-MIA cells invading the Matrigel membrane was 5.9 fold greater as compared with the MIA and EV-MIA controls (Figure 3A). These results were validated when knockdown of CD133 using CD133-specific siRNA (Supplementary Figure 1C) in CD133^hi^-MIA resulted in a significant decrease in invasion, compared with CD133^hi^-non-silencing controls (Figure 3B). As seen with stemness properties, the invasive property of clones correlated with their CD133 expression (Supplementary Figure 5C).

Consistent with the functional assay, CD133^hi^-MIA cells showed an increase in mRNA expression of several EMT-associated genes: SNAI1 (2.9 fold increase), ZEB1 (7.9 fold increase), VIM (12.5 fold increase), CDH2 (2.1 fold increase), and MMP9 (4.8 fold increase) (Figure 3C). This mesenchymal phenotype is further shown by the morphology of cells overexpressing CD133. CD133^hi^-MIA cells show a more fibroblast-like morphology, as compared with MIA and EV-MIA controls (Supplementary Figure 3A).

Another cell line, S2-VP10, derived from a liver metastasis of a patient, has demonstrated increased aggressiveness as determined by migration, invasion, and tumor progression. To confirm if these phenotypes are indeed associated with the increased CD133^+^ population in this cell line (~3%), we knocked down CD133 by CD133-specific lentivirus-shRNA in S2-VP10. Invasion decreased to 0.31 fold (Supplementary Figure 4A) in CD133^lo^-S2VP10 (0.1% CD133 surface expression) as compared with S2VP10 (3.2% CD133 surface expression) and NS-S2VP10 (3.0% CD133 surface expression) (Supplementary Figure 4B). Upon silencing of CD133 by siRNA in S2-VP10, several EMT and “stemness” related genes were downregulated (Supplementary Figure 4D). This demonstrated that the invasive phenotype of pancreatic tumor cells was dependent on their expression of CD133^+^ population.

### CD133 expression increased metastasis *in vivo*

**Figure 4:**
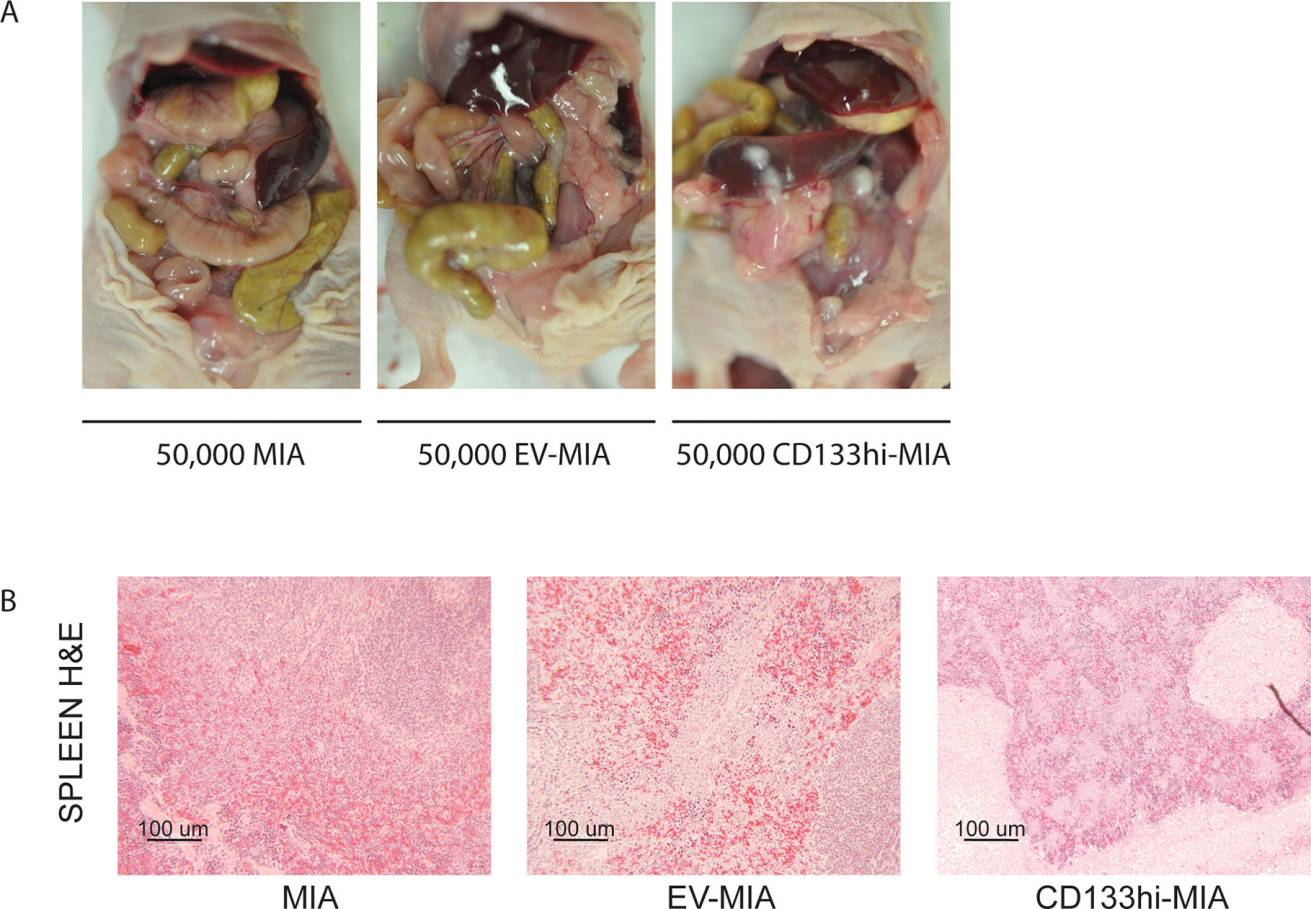
CD133 expression increased metastasis *in vivo* **(A)** Representative images of metastatic spread within the abdomen after orthotopic implantation of MIA, EV-MIA, and CD133^hi^-MIA cells. **(B)** H&E –stained sections of the spleen.

To determine the effect of CD133 expression on metastasis *in vivo*, CD133^hi^-MIA and EV-MIA cells were implanted orthotopically into the tail of the pancreas. Tumor growth was evident in all mice of each group; however, the site and spread of metastasis varied between groups (Table 2). Metastasis to spleen (4/10, 2/10, and 10/10 in MIA, EV-MIA, and CD133^hi^-MIA, respectively), lymph nodes (1/10, 0/10, and 10/10), liver (0/10, 0/10, and 6/10), abdominal wall (1/10, 0/10, and 8/10) and diaphragm (0/10, 0/10, and 5/10) increased upon overexpression of CD133 as compared with the MIA and EV-MIA controls (Table 2 and Figure 4A, 4B).

**Table 2:** CD133 overexpression led to increased metastasis *in vivo*

	MIA	EV-MIA	CD133^hi^-MIA
Spleen	4/10	2/10	10/10
Lymph Nodes	1/10	0/10	10/10
Liver	0/10	0/10	6/10
Abdominal Wall	1/10	0/10	8/10
Diaphragm	0/10	0/10	5/10

### CD133-induced invasion is mediated by activation of NF-κB

**Figure 5:**
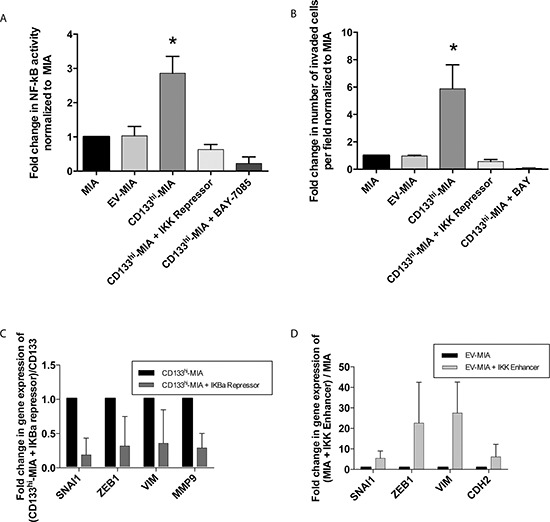
CD133 induced NF-κB activation promotes epithelial-mesenchymal transition and increases invasiveness **(A)** NF-κB activity correlated with CD133 expression and **(B)** *In vitro* invasion was decreased by NF-κB inhibition through IKK repression and pharmacological BAY 11–7085 treatment. **(C)** Decreased EMT genes upon IKBα repression in CD133^hi^-MIA cells and **(D)** induction of NF-κB activity through IKK enhancer plasmid in EV-MIA control increased EMT related genes and conversely.

NF-κB has been reported to regulate EMT and invasion [15, 16, 18]. We and others have demonstrated that CD133^+^ cells have increased activity of the NF-κB pathway as compared with CD133^−^ cells [7, 19, 20]. To determine if CD133 mediated the increased invasiveness and if EMT induction was regulated by NF-κB, we studied its activity in CD133^hi^-MIA cells using a dual luciferase assay. NF-κB activity was significantly increased in CD133^hi^-MIA cells (2.9 fold) (Figure 5A) compared with MIA PaCa-2. This increased activity further correlated with *in vitro* invasion, as determined by Boyden chamber invasion assay (Figure 5B).

Upon inhibition of NF-κB via the IKBα super repressor plasmid (S32A/S36A), decreased invasiveness was observed in CD133^hi^-MIA + IKBα-SR (0.63 fold) as compared with EV-MIA and MIA control. Similarly, inhibition of NF-κB by the pharmacological inhibitor BAY 11–7085 also showed a reduction in invasion (0.22 fold) compared with untreated cells (Figure 5B). Inhibition of NF-κB activity was confirmed via dual luciferase assay (Figure 5A). Conversely, in the S2-VP10 cell line, which has a 2–3% endogenous expression of CD133, knockdown of CD133 decreased NF-κB activity to 0.58 fold of control (Supplementary Figure 4C).

To further demonstrate that the induction of the EMT phenotype was regulated by increased NF-κB activity, a constitutively active IKK enhancer (S177E/S181E) and IKBα repressor (S32A/S36A) plasmids were utilized in the MIA control and CD133^hi^-MIA cells to increase and decrease NF-κB activity, respectively. Upon inhibition of NF-κB activity via an IKBα super repressor plasmid, expression of these EMT genes was decreased: SNAI1 (0.18 fold), ZEB1 (0.31 fold), VIM (0.35 fold), and MMP9 (0.28 fold) (Figure 5C). Conversely, upon induction of NF-κB activity by IKK enhancer plasmid within the EV-MIA control, EMT-associated gene expression increased thus: SNAI1 (5.4 fold), ZEB1 (22.6 fold), VIM (27.5 fold), and CDH2 (6.1 fold) (Figure 5D).

## DISCUSSION

For many years, CD133 has been described as a surface marker of cancer stem cells in several cancer types [5, 21, 22]. Though CD133 expression has been correlated with poor prognosis and metastasis in many different cancer types, [2, 3, 5, 6] its functional significance remains elusive.

Previous studies attempting to determine the functional relevance of CD133, have primarily manipulated cells endogenously expressing CD133 in other cancer types [4, 8, 23, 24]. Cells endogenously expressing CD133 have been comprehensively shown as a tumor-initiating cell population [3, 5, 19, 21]. Therefore, apart from expression of CD133, endogenously expressing CD133^+^ tumor initiating cells have a background providing the capacity for self-renewal, pluripotency, epithelial-mesenchymal transition, etc.

We have previously shown that cells endogenously positive for CD133, within the KPC murine model, display increased “stemness,” survival, and EMT gene expression. Additionally, CD133^+^ cells exhibited increased activation of NF-κB signaling [7]. Based on our previous findings regarding the CD133 positive population, we were interested in determining the functional relevance of this surface marker and whether its expression imparts any of the characteristics of the cancer stem cell phenotype.

This distinct background, however, may not provide the most relevant model for studying the functional role of CD133 is the cancer stem cell. To address this, we utilized a pancreatic cancer cell line with an extremely low endogenous population of CD133^+^ CSC. We were able to demonstrate how CD133 surface expression imparts to them many of the characteristics of a cancer stem cell.

Our studies showed that when overexpressed in MIA PaCa-2, as few as ten (CD133^hi^-MIA) cells, were able to form tumors in athymic mice. This indicated that CD133 expression indeed correlated with tumorigenic potential (Table 1). This increased tumorigenicity was further validated by the increased cancer stem cell phenotype that CD133 expression induced. We observed that several “stemness” genes were upregulated upon CD133 overexpression. Functionally, CD133 overexpression increased dye efflux and ALDH activity, characteristics described for authentic cancer stem cells [25, 26].

Apart from tumor initiation, many CSC populations represented by the surface expression of CD133 showed increased metastatic potential [13, 27]. Additionally, circulating tumor cells are also enriched in CSC markers [28]. Thus far, two CSC populations in pancreatic cancer have been shown to be metastatic, CD133^+^CXCR4^+^ [3] and Met^+^CD44^+^ [29]. CD133^+^CXCR4^+^ cells derived from an immortalized pancreatic tumor cell line were shown to metastasize *in vivo* as compared with the CD133^+^CXCR4^−^ cell subset. In addition, the Met^+^CD44^+^ CSC population was also capable of metastasis, which was abrogated upon pharmacological Met inhibition.

Our studies further demonstrated that the increased invasiveness was dependent on CD133 surface expression, as surface expression correlated with *in vitro* invasion. CD133 overexpression in an endogenously low-expressing CD133 cell line, MIA PaCa-2, increased cellular invasiveness (Figure 3A). Additionally, using both siRNA and lentiviral knockdown of CD133 in CD133^hi^-MIA (Figure 3B) or the cell line with endogenously high CD133^+^ population, S2VP10 (Supplementary Figure 4A) respectively, we observed a correlation between surface expression and invasiveness. From these data we concluded that invasive potential is dependent on CD133 surface expression.

In order to invade, cells induce the epithelial-mesenchymal transition (EMT) process required to detach from the primary tumor, migrate, and embed at a distant site. We next examined whether overexpression of CD133 would influence the gene expression of several EMT-associated genes. We saw a significant upregulation in several key EMT transcription factors (SNAI1 and ZEB1) and other EMT-associated markers (VIM, CDH2, MMP9) (Figure 3C). These data suggest that induction of CD133 induced EMT-associated gene expression contributs to their increased invasiveness.

To demonstrate metastatic potential *in vivo*, we implanted MIA, EV-MIA, and CD133^hi^-MIA cells into the pancreas of athymic mice. We found sizable primary tumors in all groups. However, tumors derived from CD133^hi^-MIA cells demonstrated local invasion to the spleen and metastasis to the spleen, lymph nodes, liver abdominal wall, and diaphragm at a greater rate than in controls (Table 2), while minimal metastasis was observed in the MIA and EV-MIA control groups. This indicated that cells expressing CD133 are able to invade and metastasize better than cells lacking CD133 expression. CD133 therefore seems to be responsible for at least part of the CSC's ability to preferentially metastasize over its marker-negative subset.

The process of invasion is regulated by NF-κB-mediated signaling [15, 16, 18]. We [7] and others [19, 20] have shown that endogenously expressing CD133^+^ cells have increased NF-κB activity compared with the CD133^−^ subset. In our study, NF-κB activity correlated with *in vitro* invasion and the inhibition of NF-κB signaling through IKK repressor plasmid or BAY 11–7085 decreased this invasiveness (Figure 5A and 5B). Therefore, we concluded that the increased invasiveness imparted by CD133 expression was reliant on NF-κB activation.

Many EMT genes are regulated by the NF-κB pathway. Our data showed that activation of NF-κB activity through IKK enhancer plasmid increased EMT-associated gene expression (Figure 5D) that was similar to that observed with CD133 overexpression. Conversely, repression of NF-κB activity in CD133^hi^-MIA cells significantly decreased expression of these genes (Figure 5C). This showed that EMT induction in CD133 positive cells was dependent on NF-κB activity.

Corroborating our findings, other studies in differing cancer types demonstrated that the silencing of CD133 within endogenously-expressing CSCs decreases “stemness” as shown by tumor initiation, as well as decreased invasion and metastasis [8, 24]. These studies complement our work by revealing that CD133 expression within the context of an authentic CSC is essential for the “stemness” and metastatic potential of this population.

In conclusion, these data convey the importance of CD133, a CSC surface marker, for the biology of the CSC. CD133 was once viewed as merely a surface glycoprotein characteristic of this population. In this study we demonstrate that CD133 expression influences tumor initiation, progression, and metastasis. We determined that the increased metastatic potential of CD133-expressing cells is mediated by the induction of NF-κB pathway activation. NF-κB activation by CD133 surface expression increased cellular invasion and induced EMT. These data indicate that CD133 contributes significantly to the phenotype of this important cancer stem cell population.

## MATERIALS AND METHODS

### Generation of stable cell lines

MIA PaCa-2 (ATCC) and stable MIA-derivatives were maintained in DMEM (Hyclone) containing 10% fetal bovine serum. S2-VP10 cells were cultured in RPMI 1640 (Hyclone) containing 10% fetal bovine serum. Stable clones were selected and maintained in Geneticin (Invitrogen) and Puromycin (Clontech) for MIA PaCa-2 and S2-VP10 derivatives, respectively.

### Plasmids and vectors

Human cDNA CD133 expression plasmid (EX-Z0396-M02) and empty vector plasmid (EX-NEG-M02) were obtained from GeneCopoeia. Lentiviral shRNA pGIPZ vectors; NS (RHS4348) and αCD133 (V2LHS_71816) were obtained from Thermo Scientific. IKK (IKK-2 S177E S191E) and IKBα (pBabe-Puro-IKBalpha-mut (superrepressor)) plasmids were obtained from Addgene.

### Boyden chamber invasion assay

24-well transwell inserts (BD Biocoat) were hydrated using serum-free media and 25,000 cells were plated into the top chamber of the inserts in serum-free media with 10% serum medium in the bottom chamber. After 24 hours, cells in the top chamber were removed by scrubbing with a cotton swab, fixed in methanol, and stained using crystal violet. Cells having migrated through the Matrigel were counted by microscopy and compared with controls to determine the change in invasiveness.

### NF-κB activity assays

NF-κB activity was determined by both p50 binding ELISA (Thermo Scientific) and Dual-Luciferase reporter assay (Qiagen). Binding ELISA was performed according to the manufacturer's protocol using whole cell lysates and values were normalized to μg protein as determined by protein estimation (Pierce). Dual-Luciferase reporter assay system (Promega) results were determined by Synergy2 luminometer (Biotek).

### Flow cytometric assays

All FACS analyses were performed on the BD FACSCanto II (BD Biosciences) using analysis software FACSDiva (BD Biosciences) and FlowJo (Tree Star). Debris and cell clusters were excluded from analysis by side- and forward-scatter adjustments. Aldefluor assay was performed by manufacturer's instructions (Stem Cell Technologies) complete with DEAB controls. Drug efflux assay was performed by adding Nucblue live cell reagent (Life Technologies) to live cells with or without verapamil treatment. Drug efflux activity was determined by analysis of the extent of the shift in the population.

### Animals and tumor xenografts

Female, athymic nude mice were purchased from the National Cancer Institute and experiments were carried out according to Institutional Animal Care and Use Committee-approved protocols. Cells were injected into both flanks of each mouse using Matrigel:media (1:1) at 10 and 1000 cells per flank. CD133^hi^-MIA cells were magnetically sorted (Miltenyi Biotec) for CD133 prior to injection. Mice were assessed biweekly for tumor formation. For the orthotopic xenograft model, animals were anesthetized, and a left lateral incision was given with a scalpel. The abdominal cavity was opened using scissors, and pancreatic cancer cells were injected into the pancreas. The abdominal cavity was closed by absorbable suture and skin closed using staples. Tumor formation was observed weekly by palpation. The mice were sacrificed at 5 weeks to determine the extent of metastasis.

### Histology

Animals were sacrificed and tissues were resected. Tumor, spleen, liver, and lungs were dissected for histopathological examination. Briefly, tissue specimens were fixed in 10% formalin followed by 80% ethanol, paraffin-embedded, sectioned, and stained with hematoxylin and eosin for histological analysis.

### Statistical Methods

Values are expressed as mean ± SEM. *In vitro* experiments were performed a minimum of three times and significance between two samples was determined using one way ANOVA analysis. Values were considered statistically significant when *p* < 0.05.

## SUPPLEMENTARY FIGURES


