# Supplementary material for: CD133 initiates tumors, induces epithelial-mesenchymal transition and increases metastasis in pancreatic cancer
Source: Oncotarget. 2015 Mar 16;6(10):8313–22. doi: 10.18632/oncotarget.3228 (PMC4480754; doi:10.18632/oncotarget.3228)
Supplement: Supplementary file 1 [file oncotarget-06-8313-s001.pdf]

SUPPLEMENTARY FIGURES

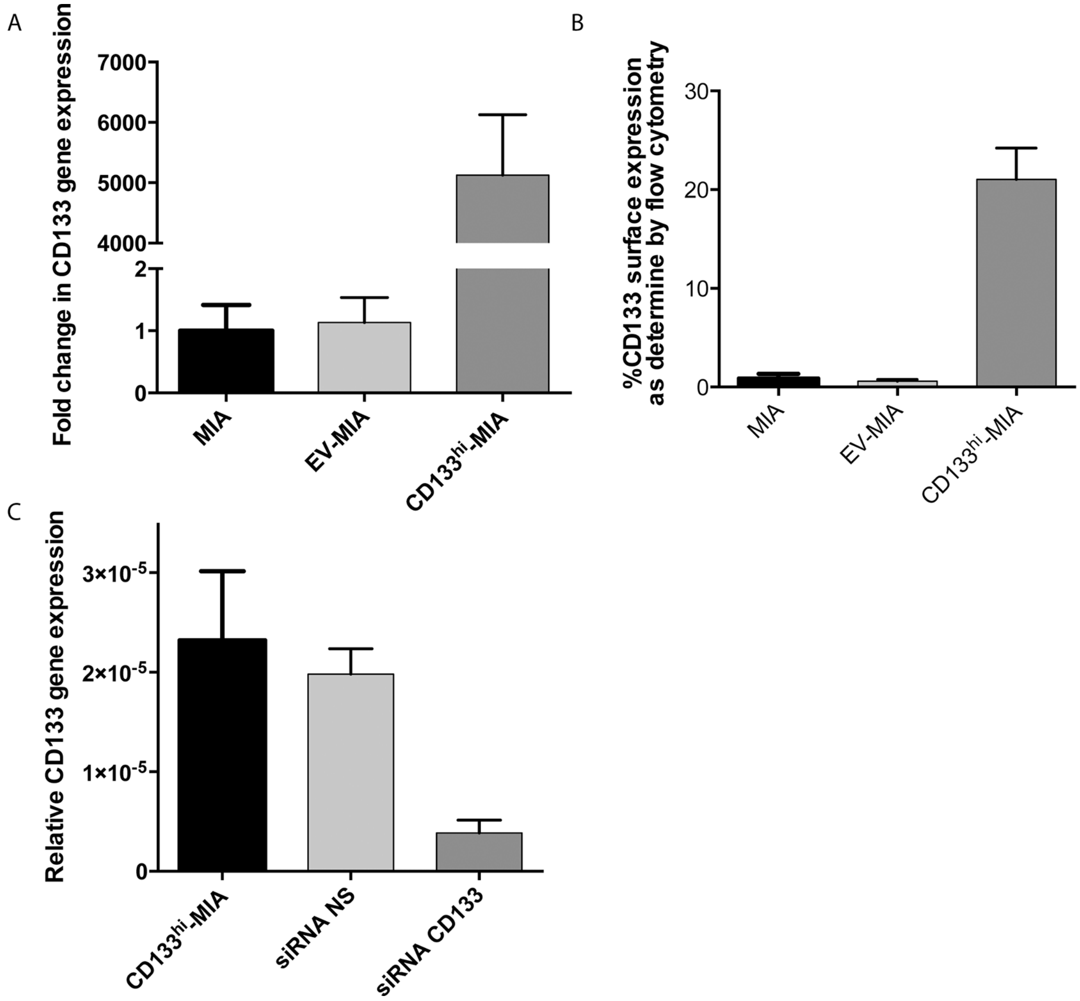

Supplementary Figure 1: CD133 overexpression in stable cell lines. (A) Gene expression in MIA, EV-MIA, and CD133^{hi}-MIA stable cell lines, **(B)** surface CD133 expression as determined by flow cytometry, and **(C)** CD133 gene expression upon CD133 siRNA silencing.

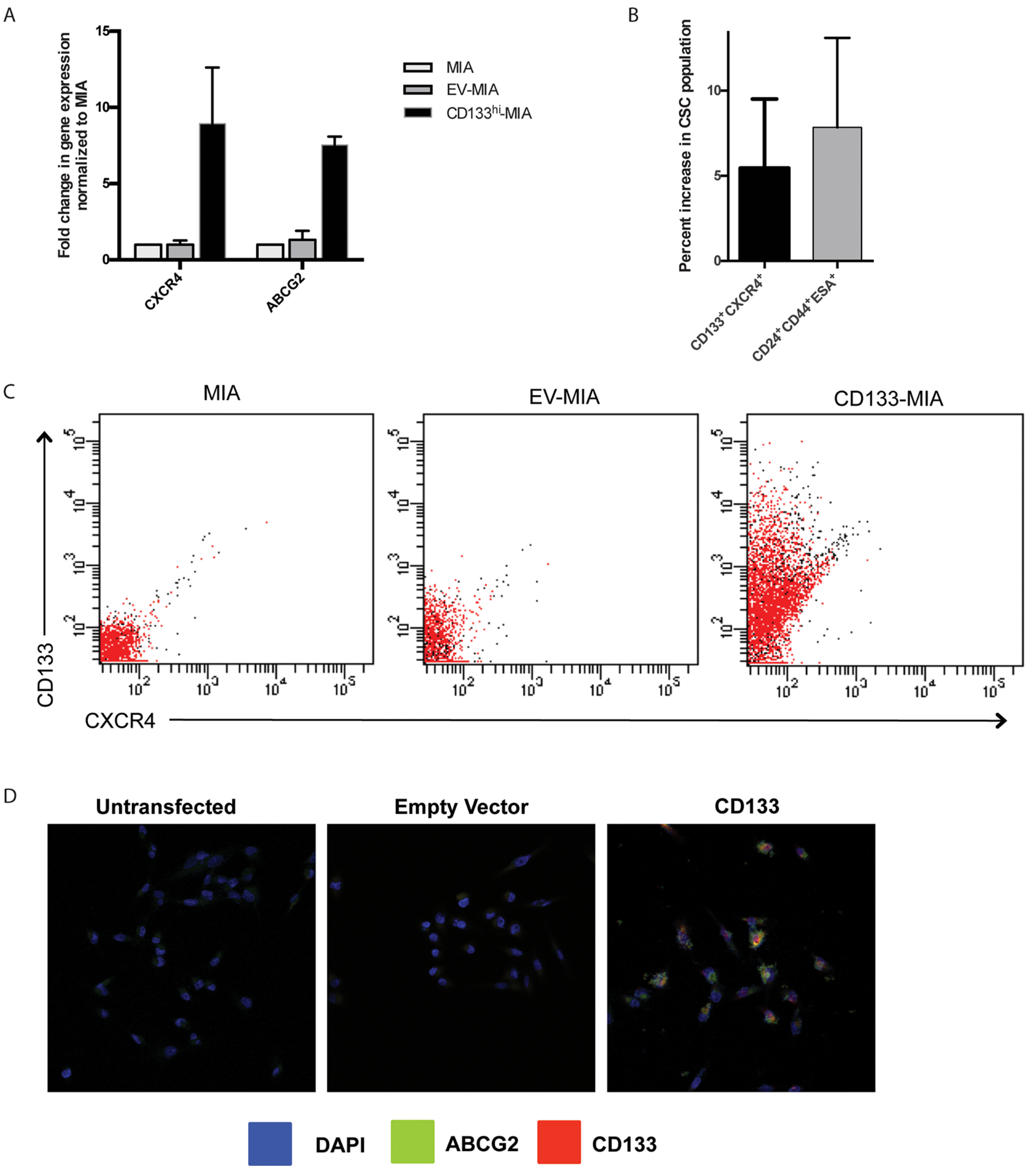

Supplementary Figure 2: CD133 overexpression increases cancer stem cell markers. **(A)** RNA expression of CXCR4 and ABCG2, **(B)** surface expression of CD133 and CXCR4 by flow cytometry, and **(C)** surface expression of ABCG2 by immunofluorescence.

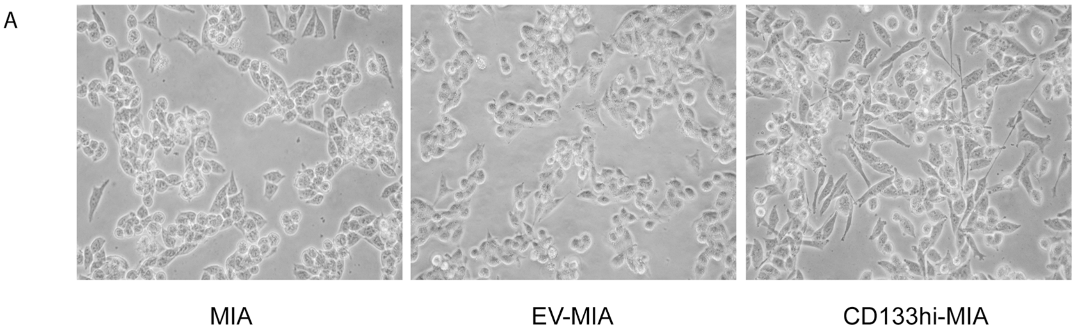

Supplementary Figure 3: CD133 overexpression induces a more fibroblast-like morphology. **(A)** Representative images of MIA, EV-MIA, and CD133^{hi}-MIA cell lines.

A

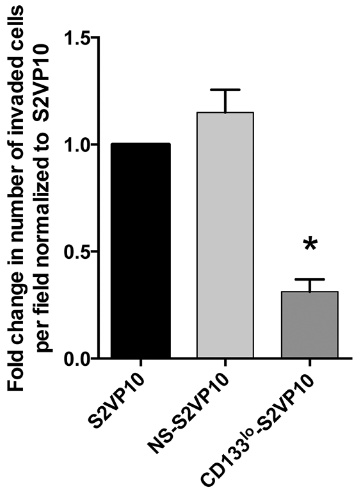

B

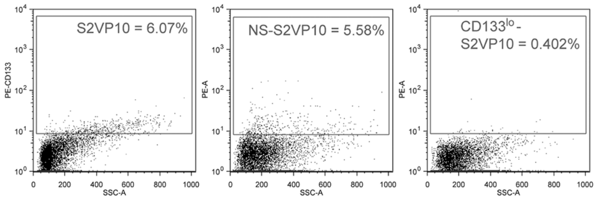

C

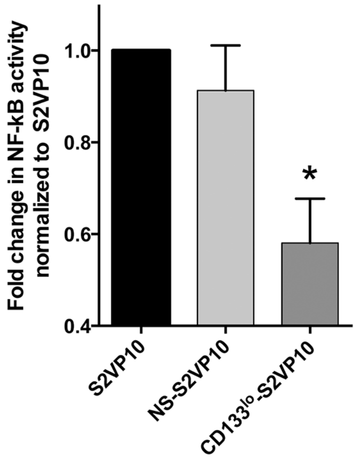

D

Epithelial-Mesenchymal Transition related genes	
	S2VP10 CD133 siRNA (fold change over NS)
**SNAI1**	0.408 (± 0.021)
**SNAI2**	0.552 (± 0.024)
**TWIST**	0.141 (± 0.072)
**ZEB1**	0.328 (± 0.060)
**VIM**	0.450 (± 0.044)
**CDH2**	0.566 (± 0.126)
**MMP7**	0.265 (± 0.040)
**MMP9**	0.264 (± 0.043)

"Stemness" related genes	
	S2VP10 CD133 siRNA (fold change over NS)
**KITLG**	0.481 (± 0.019)
**LIN28B**	0.544 (± 0.070)
**GLI1**	0.347 (± 0.061)
**SOX2**	0.460 (± 0.007)
**NANOG**	0.598 (± 0.086)
**SIRT1**	0.492 (± 0.020)
**POU5F1**	0.710 (± 0.083)
**ALDH1**	0.212 (± 0.017)

Supplementary Figure 4: Silencing CD133 decreases invasion and NF-κB activity. (A) *In vitro* invasion represented as fold change normalized to S2-VP10 control compared to LV-shRNA-αCD133 knockdown cell lines. **(B)** CD133 surface expression as determined by flow cytometry. **(C)** NF-κB activity in S2-VP10 and shRNA derivatives by dual-luciferase assay. **(D)** Gene expression changes in EMT and "stemness" related genes upon silencing of CD133.

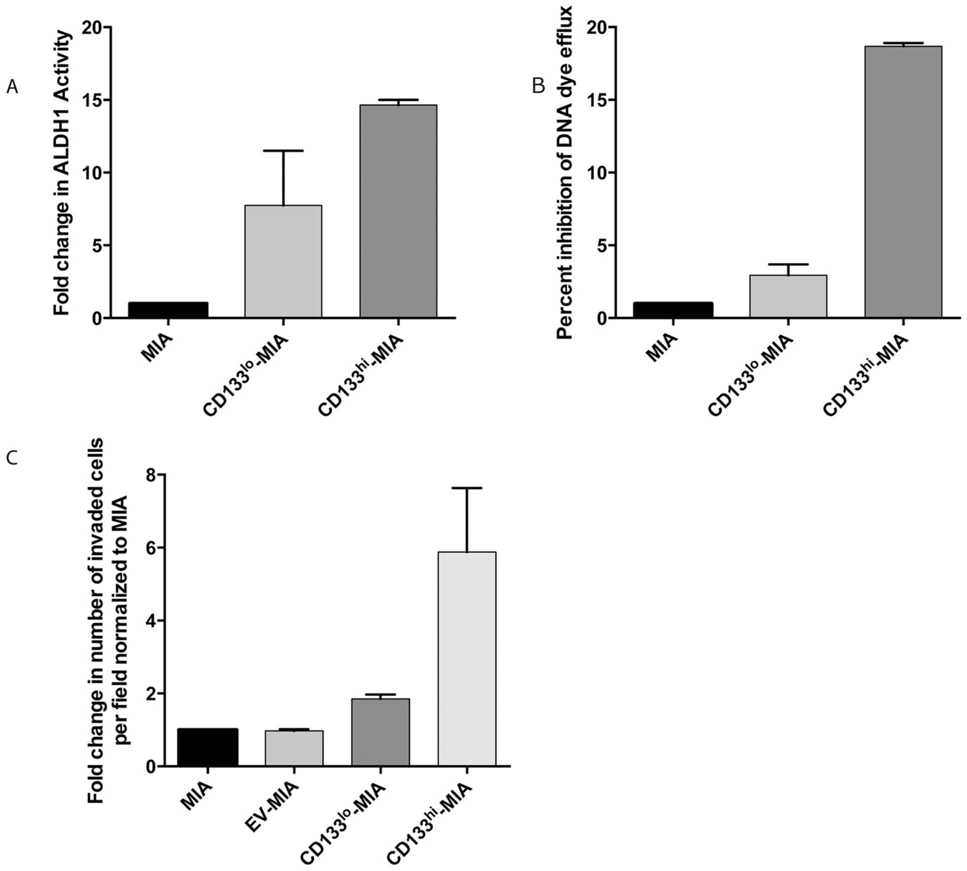

Supplementary Figure 5: Degree of CD133 surface overexpression correlates with "stemness" and invasion. (A) ALDH1 activity as measured by flow cytometry, **(B)** Dye efflux potential as determined by flow cytometry, and **(C)** *in vitro* invasion by Boyden chamber invasion assay.
